# Cardiovascular magnetic resonance of cardiomyopathy in limb girdle muscular dystrophy 2B and 2I

**DOI:** 10.1186/1532-429X-13-39

**Published:** 2011-08-04

**Authors:** Xiomara Q Rosales, Sean J Moser, Tam Tran, Beth McCarthy, Nicholas Dunn, Philip Habib, Orlando P Simonetti, Jerry R Mendell, Subha V Raman

**Affiliations:** 1 Center for Gene Therapy, The Research Institute at Nationwide Children's Hospital, Columbus, Ohio 43205, USA; 2 The Ohio State University, Department of Pediatrics and Neurology, Columbus, Ohio 43210, USA; 3 The Ohio State University, Davis Heart and Lung Research Institute, Columbus, Ohio 43210, USA

## Abstract

**Background:**

Limb girdle muscular dystrophies (LGMD) are inclusive of 7 autosomal dominant and 14 autosomal recessive disorders featuring progressive muscle weakness and atrophy. Studies of cardiac function have not yet been well-defined in deficiencies of dysferlin (LGMD2B) and fukutin related protein (LGMD2I). In this study of patients with these two forms of limb girdle muscular dystrophy, cardiovascular magnetic resonance (CMR) was used to more specifically define markers of cardiomyopathy including systolic dysfunction, myocardial fibrosis, and diastolic dysfunction.

**Methods:**

Consecutive patients with genetically-proven LGMD types 2I (n = 7) and 2B (n = 9) and 8 control subjects were enrolled. All subjects underwent cardiac magnetic resonance (CMR) on a standard 1.5 Tesla clinical scanner with cine imaging for left ventricular (LV) volume and ejection fraction (EF) measurement, vector velocity analysis of cine data to calculate myocardial strain, and late post-gadolinium enhancement imaging (LGE) to assess for myocardial fibrosis.

**Results:**

Sixteen LGMD patients (7 LGMD2I, 9 LGMD2B), and 8 control subjects completed CMR. All but one patient had normal LV size and systolic function; one (type 2I) had severe dilated cardiomyopathy. Of 15 LGMD patients with normal systolic function, LGE imaging revealed focal myocardial fibrosis in 7 (47%). Peak systolic circumferential strain rates were similar in patients vs. controls: ε_endo _was -23.8 ± 8.5vs. -23.9 ± 4.2%, ε_epi _was -11.5 ± 1.7% vs. -10.1 ± 4.2% (p = NS for all). Five of 7 LGE-positive patients had grade I diastolic dysfunction [2I (n = 2), 2B (n = 3)]. that was not present in any LGE-negative patients or controls.

**Conclusions:**

LGMD2I and LGMD2B generally result in mild structural and functional cardiac abnormalities, though severe dilated cardiomyopathy may occur. Long-term studies are warranted to evaluate the prognostic significance of subclinical fibrosis detected by CMR in these patients.

## Background

Limb-girdle muscular dystrophy (LGMD) comprises a group of genetically-heterogeneous disorders that present with variable skeletal and cardiac muscle involvement[1-3]. LGMD produces progressive weakness of proximal shoulder-girdle or pelvic muscles with a wide range of phenotypic expression, severity, and age of disease onset[4-6]. In other dystrophies, the degree of cardiac involvement is directly related to life expectancy,[7] and cardiac muscle manifestations must be addressed, as treatment options are evolving for skeletal muscle[8]. LGMD2B is part of the family of dysferlin deficient myopathies inherited as autosomal recessive disorders. Dysferlin is an important component of the muscle membrane repair system, which forms vesicle plugs over membrane lesions, maintaining cell homeostasis critical to the formation of new membrane following sarcolemmal injuries[9]. LGMD2B represents one end of the clinical spectrum of the dysferlinopathies[9,10]. In contrast, another major dysferlin-deficient phenotype is Miyoshi myopathy, presenting with distal lower limb weakness and atrophy affecting the posterior calf muscles[9,10]. Clinical experience, however, suggests that there is significant overlap in presentation of these presumably distinct entities[11-13]. Dysferlin deficiency represented the most common abnormality in our North American LGMD characterization study[14] and this was also true of a European cohort[1]. Cardiac involvement has not been systematically studied in dysferlin deficiency[15]. In a small cohort, Wenzel *et al. *observed symptomatic dilated cardiomyopathy in 2 of 7 total subjects[16].

The autosomal recessive subtype LGMD-2I is caused by a mutation in the fukutin-related protein gene (FKRP), which is thought to encode for a protein involved in α-dystroglycan glycosylation[5]. This particular subtype represents another one of the more common forms of LGMD in North America[14,17]. Previous studies have estimated a broad range of cardiac involvement (10-60%) in subjects with LGMD2I [15,18,19]. Further, the degree of cardiomyopathy does not correlate with the severity of skeletal myopathy[15]. Given that cardiovascular magnetic resonance (CMR) is the most discriminating methodology to characterize cardiac structure and function, we sought to better characterize the myocardium in LGMD2I and 2B for several reasons. For example, some patients may warrant cardioprotective therapy if CMR abnormalities are observed at a time when systolic function is preserved by echocardiography, though this is not current practice in LGMD unlike in Duchenne muscular dystrophy (DMD) where angiotensin-converting enzyme (ACE) inhibitors are often started when EF is normal by echocardiography yet CMR reveals abnormalities in strain and evident myocardial fibrosis[20-22]. In addition, when considering patients for clinical trials, knowledge of predilection toward cardiomyopathy could influence protocol design for cardiac monitoring to reduce drug-related cardiotoxicity.

## Methods

### Subjects

Sixteen subjects with confirmed LGMD2I (n = 7) or LGMD2B (n = 9), all with predominantly proximal weakness or combined proximal and distal weakness, were prospectively enrolled through a National Institutes of Health (NIH)-supported LGMD characterization study (NIAMS U54 AR050733-05) from October 2006 until January 2009. The study design was approved by the Institutional Review Board, and all subjects gave written informed consent to participate. Two additional subjects had severe claustrophobia, ferromagnetic metals, or other devices not compatible with magnetic resonance precluding enrollment. Consistent information was obtained for each patient to establish ethnic and geographical origin, family history, consanguinity, age at onset, initial distribution of symptoms, pattern of muscle involvement, ambulatory status, disease progression, and serum creatine kinase (CK) levels.

### Genetic analysis

Genomic DNA was isolated from peripheral blood from each patient (Pure-Gene; Qiagen), as previously described[23] for sequence analysis of the FKRP and/or dysferlin gene.

### CMR

All subjects underwent comprehensive CMR examination using a 1.5 Tesla clinical scanner (Siemens MAGNETOM Avanto). The CMR protocol included: horizontal long-axis, vertical long-axis, and three chamber long-axis views, in addition to continuous short-axis cine true-FISP acquisition to measure right and left ventricular volumes, and ejection fraction (EF); multiplane late gadolinium enhancement imaging (LGE) was performed using a fast gradient echo inversion-recovery prepped technique with appropriate inversion time selection[24]. Transmitral flow measurements were obtained at the mitral leaflet tips in end-diastole using electrocardiographically-triggered phase-contrast acquisition with a velocity sensitivity of 130 cm/s; myocardial tissue velocities were measured phase contrast MR prescribed at the basal third of the LV in a short-axis plane with velocity encoding of 30 cm/s[25].

### Image Analysis

Image analysis was performed offline by investigators who were blinded to the subject's history. LV EF, volumes and mass were measured from short axis contiguous cine images using semi-automated delineation of endocardial and epicardial contours at end-diastole and end-systole and Simpson's rule[26]. Peak systolic endocardial circumferential strain (ε_cc_) was derived from mid-short axis cine images using semi-automated Vector Velocity Imaging (VVI) feature tracking software (Siemens, Mountain View, CA). Initially developed for segmentation of echocardiographic images through speckle tracking[27], the software similarly applies feature tracking of the myocardium yielding endocardial and epicardial borders from cine CMR data through the cardiac cycle from manually drawn contours on a single image frame. Endocardial circumferential strain derived by this method has been validated against tagged MRI harmonic phase analysis (HARP) in a population of patients with Duchenne muscular dystrophy[28]. No segmental analysis was performed as only whole-slice ε_cc _measurements have been previously validated.

Myocardial pixels were automatically followed over time to generate velocity vectors of length L, with initial length referred to as L_0_. The peak systolic strain rate in the circumferential direction (ε_cc_) for the endocardium and epicardium were calculated from the formulae: strain = (L-L_0_)/L_0_, and strain rate = strain/time. The presence of myocardial fibrosis was assessed by reviewing the multiplane LGE images as present or absent if signal intensity in regions of hyperenhancement exceeded 2SD above the mean signal intensity in normal myocardium. Classification of diastolic function as normal, grade I diastolic dysfunction (impaired relaxation), grade II dysfunction (pseudonormal), and grade III dysfunction (restrictive filling pattern) was based on mitral inflow velocity patterns according to standard criteria[29-31].

### Statistical Analysis

Continuous variables are expressed as mean ± standard deviation (SD). Volumes and mass are expressed relative to body surface area. The mean values of continuous variables were compared using two-sample *t*-test, and correlation between continuous variables was computed with the Pearson coefficient.

## Results

### Clinical Features

Clinical features of study subjects are summarized in Table 1. In the LGMD2B cohort (six men, three women), the mean age of onset of muscle weakness was 16 years of age (range 3-30 years). Five of the nine subjects became wheelchair-dependent at a mean age of 38 years (range 28-46). In the FKRP cohort (three men, four women), the mean age of onset was 11 years (range 3-18 years). One of the LGMD2I patients had late onset muscle disease at age 48 accompanied by a severe dilated cardiomyopathy (patient 6 in Table 1). Concurrent illnesses included 7 subjects with hypertension (2 with LGMD2I and 5 with LGMD2B), three LGMD2B with type 2 diabetes mellitus, and one LGMD2B with coronary artery disease.

**Table 1:** Phenotypic features

	Clinical Data							
**Patient**	**Diagnosis**	**Sex/Age**	**Age of onset**	**Onset mode**	**ALA**	**CK**	**MRI findings**	**Comorbidity**

1	FKRP	F/44	08	PLE	Amb.	NA		
2	FKRP	M/64	14	PLE	30	NA	diastolic dysfunction, myocardial fibrosis	HTN
3	FKRP	F/43	18	PLE	Amb.	4486		
4	FKRP	F/20	10	PLE	Amb	NA	myocardial fibrosis	
5	FKRP	M/11	3	PLE	Amb	8000	myocardial fibrosis	
6	FKRP	M/58	48	PLE	Amb	NA	severe systolic dysfunction, myocardial fibrosis	HTN
7	FKRP+SGCA	F/24	12	PLE	Amb	2071		
8	DYSF	M/49	30	DUE+PLE	Amb	3481		
9	DYSF	M/43	17	DLE	28	7938		DM,HTN
10	DYSF	M/48	25	PLE	46	9468	diastolic dysfunction	DM,HTN
11	DYSF	M/51	03	PLE	Amb	280	diastolic dysfunction, myocardial fibrosis	DM,CAD
12	DYSF	M/47	6	PLE	43	428	diastolic dysfunction, myocardial fibrosis	
13	DYSF	M/54	14	DLE	45	1911	diastolic dysfunction, myocardial fibrosis	HTN
14	DYSF	F/43	19	PLE	Amb	1234		HTN
15	DYSF	F/44	16	DLE	31	320		HTN
16	DYSF	F/33	19	PLE	Amb	3046		

FKRP, Fukutin-related protein; SGCA, alpha-Sarcoglycan gene; DYSF, dysferlin gene; CK, creatine kinase; DLE, distal lower extremity; DUE, distal upper extremity; PLE, proximal lower extremity; PUE, proximal upper extremity; ALA, age lost ambulation; Amb, ambulatory;HTN, hypertension; DM, diabetes mellitus, CAD, coronary artery disease; NA, no available.

### Genetic Analysis

The distribution of mutations for both LGMD2B and 2I is summarized in Table 2. Five of the seven LGMD2I (FKRP) subjects were homozygous for the common missense mutation [826C>A], one has the same mutation [826C>A] detected at only one allele and another patient exhibited heterozygous missense mutations [c.520A>T] + [c.-34C>T] previously reported in the literature and Leiden database[32,33]. Heterozygous mutations predicted a more severe course as previously described[34,35]. Of interest, one mutation proven FKRP subject (Table 2, patient 7) was also found to also have a known pathogenic mutation [850C>T] at one allele in the alpha-sarcoglycan gene. This mutation is one of the two pathogenic mutations found in this patient's family (three aunts and one uncle affected with LGMD2D). We could not distinguish any identifiable effect on the patient's FKRP phenotype from this alpha-sarcoglycan carrier allele.

**Table 2:** Pathogenic mutations in the 16 patients.

Patient	Gene	Mutation
1	FKRP	[c.826C>A]+ [c.826C>A]
2	FKRP	[c.520A>T]+ [c.-34C>T]
3	FKRP	[826C>A]+ [826C>A]
4	FKRP	[826C>A]+ [826C>A]
5	FKRP	[826C>A]
6	FKRP	[826C>A]+ [826C>A]
7	FKRP+SGCA	[826C>A]+[826C>A] in FKRP + [850C>T] in SGCA gene*
8	DYSF	[c.2643+1G>A]+[c.4577A>C]
9	DYSF	[c.1481-1G>A]+[c.5836_5839del]
10	DYSF	[c.3892A>G]
11	DYSF	[c.3892A>G]
12	DYSF	[c.3065G>A; c.3992G>T]
13	DYSF	[c.610C>T]+ [c.5884C>T]
14	DYSF	[c.2408G>A]
15	DYSF	[c.1392dupA]+[c.3516_3517delTT]
16	DYSF	[c.1120G>C]+[c.5713C>T]

FKRP, Fukutin-related protein; SGCA, alpha-Sarcoglycan gene; DYSF, dysferlin gene. *Patient #7 was found homozygous for a common mutation [826C>A] in the FKRP gene, and also carrier of one known pathogenic mutation [850C>T] in the alpha-sarcoglycan gene (LGMD type 2D) in a heterozygous state.

Mutations in the dysferlinopathy cohort were distributed as follows: 7 missense, 2 splice-site, 3 nonsense resulting in premature termination codons, and 3 frame-shift mutations. In 3 of the nine dysferlinopathy patients (#10, #11, and #14) only one mutation was identified; patient #10 and #11 presented with a common mutation [c.3892A>G] previously described as pathogenic in several articles in the literature and also reported in the Leiden database[11,36,37]. This mutation was not detected in at least 100 chromosomes reported in the Nature Genet article (Nat Genet 1998; 20:31-36) [11]. Patient #14 presented with one missense mutation not previously reported but indicative of a pathogenic effect based on bioinformatics analyses (PolyPhen and UMDpredictor).

### CMR

The LGMB2I patient with severe LV enlargement and systolic dysfunction (patient 6) had EF 12%, end-diastolic volume (EDVI) 217 mL/m^2 ^and end-systolic volume (ESVI) 191 ml/m^2^. LV EF was normal in the remaining subjects, averaging 60 ± 7%, and these patients had normal cardiac volumes (EDVI 55 ± 15 mL/m^2^, ESVI 23 ± 8 mL/m^2^). Also, all patients excluding the subject with dilated cardiomyopathy had relatively normal LV mass, which averaged 53 ± 19 g/m^2^. Systolic circumferential strain analysis also showed no difference between patients and controls (Table 3).

**Table 3:** Circumferential Strain in LGMD Patients vs. Controls

	LGMD Patients (N = 16)	Controls (N = 8)
Peak circumferential strain of the endocardium	-23.8 ± 8.5%	-23.9 ± 4.2%
Peak circumferential strain of the epicardium	-11.5 ± 1.7%	-10.1 ± 4.2%

**Figure 1:**
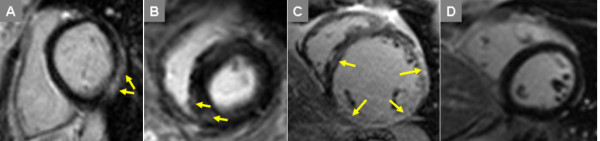
**LGE Findings in LGMD**. Late post-gadolinium enhancement imaging in LGMD patients typically demonstrated focal epicardial (A) or midwall (B) enhancement; one patient with LGMD2I and advanced dilated cardiomyopathy had extensive myocardial injury/fibrosis (C). Control subjects showed no myocardial enhancement on LGE imaging (D).

LGE imaging showed extensive myocardial fibrosis in one LGMB2I patient with advanced cardiomyopathy (patient 6). In the remaining 15 LGMD patients, seven (47%) had focal enhancement not present in controls. Prevalence within specific genotypes was 4 of 7 (57%) patients with LGMD2I, and 3 of 9 (33%) patients with LGMD2B. Patterns of enhancement included epicardial, similar to the pattern seen in dystrophin-deficient cardiomyopathy and some myocarditis, as well as the midwall fibrosis of other nonischemic cardiomyopathies (Figure 1).

Mitral inflow velocities revealed E-A reversal, consistent with grade I diastolic dysfunction in 5 LGE-positive patients (4 type 2B, 1 type 2I); diastolic function was normal in the remaining patients and in all controls.

## Discussion

The majority of patients with LGMD of 2 subtypes - 2B, and 2I - in our cohort showed normal LV size, global systolic function and peak systolic circumferential strain. However, there was evidence of subclinical myocardial fibrosis in 57% of subjects with LGMD2I and 33% of subjects with LGMD2B. This abnormality was accompanied by diastolic dysfunction in 5 patients [2I (n = 1), 2B (n = 4)]. The combination of these findings is consistent with our previous observations and those of others demonstrating a link between myocardial fibrosis and diastolic dysfunction irrespective of etiology[38,39]. Subjects with LGMD2I displayed a high degree of myocardial fibrosis with preserved systolic function. It is unclear whether or not the advanced dilated cardiomyopathy seen in one LGMD2I subject was primarily caused by the genetic disorder or if it was an acquired condition, perhaps compounded by genetic susceptibility.

There have been two prior studies evaluating myocardial abnormalities with CMR in subjects with LGMD2I. Gaul *et al. *studied 9 subjects, 8 of whom had reduced LVEF, increased LV mass, or increased LV end-diastolic volume[40]. Diastolic function was evaluated by echocardiography and was found to be abnormal in two subjects[40]. The study did not include LGE imaging to evaluate for myocardial fibrosis, a technique now considered a *sine qua non *in the CMR evaluation of cardiomyopathy[41]. Wahbi *et al. *characterized myocardial involvement in 11 of 13 LGMD2I patients with LGE-CMR, and found a high incidence of LV and RV myocardial fibrosis in addition to fatty replacement[18]. It is uncertain if the high prevalence of fibrosis would persist after applying the more rigorous quantitative threshold used in our work.

While previous work in muscular dystrophy-associated myocardial disease has similarly applied processing of routinely-acquired cine imaging to successfully derive strain parameters[28], much work has also been done using specialized techniques for strain measurement. Using tagged cine CMR, Hor *et al. *found significant differences in systolic circumferential strain between boys with DMD and controls, even in DMD patients with preserved ejection fraction[20]. Similarly, Smith *et al. *identified abnormal circumferential strain in patients with autosomal dominant Emery-Dreifuss muscular dystrophy due to lamin A/C mutation[42]. Not finding such a difference in LGMD2B patients suggests that much remains to be elucidated as to how distinct myocyte protein genotypes produce such a broad spectrum of phenotypes[43]. Measures of other aspects of cardiac mechanics such as torsion may be revealing as well[44,45].

To our knowledge, our study represents the largest evaluation using CMR of LGMD2B patients. It has been proposed that dysferlin promotes cardiomyocyte repair, and that dysferlin deficiency leads to cardiomyopathy[46,47]. Diastolic dysfunction and myocardial fibrosis were common in this form of limb girdle muscular dystrophy. The extent to which concomitant hypertension contributes to fibrosis in these patients cannot be determined, though of 7 with fibrosis only 3 had hypertension.

A study of 100 subjects with LGMD2A to 2I used echocardiographic and electrocardiographic criteria to define cardiac involvement (CI): EF <50%, intraventricular septum greater than 1.2 cm, left bundle branch block, atrial fibrillation/flutter, or atrioventricular conduction block[15]. Using these criteria, 24% had CI including 29% of those with LGMD2I and one of the two subjects with LGMD2B. Future studies that combine LGE data should help define the myocardial substrate for these structural and functional abnormalities.

## Conclusions

The prevalence of advanced cardiomyopathy in patients with LGMD2I and LGMD2B appears to be limited, but subclinical fibrosis and diastolic dysfunction do occur and may warrant institution of cardioprotective medical therapies. The combination of skeletal and cardiac muscle involvement, more common in LGMD2I, point clinicians in the direction of this genotype when faced with an unknown form of limb girdle muscular dystrophy. Longitudinal studies are warranted to define the natural history of LGMD patients with subclinical abnormalities in cardiac structure and function.

## Competing interests

The authors declare that they have no competing interests.

## Authors' contributions

XQR, JRM and SVR conceived of the study, and XQR directed enrollment and collection of clinical data. SVR directed implementation of the CMR studies and analysis of data. OPS supervised and SJM and ND performed strain analysis. PH contributed to literature review and manuscript preparation. TT assisted with image analysis. BMc contributed to study coordination. All authors read and approved the final manuscript.
